# Occurrence, source identification, and human health risk assessment of potentially toxic elements in freshwater sources used for aquaculture in the Senegal, Gambia, and Casamance river basins

**DOI:** 10.1016/j.toxrep.2026.102340

**Published:** 2026-09-17

**Authors:** Safiétou Mbaye, Dani Khoury, Gaspard Conseil, Jean Fall, Pamela Hartmeyer, Frédéric Pierlot, Damien Banas

**Affiliations:** a University Institute of Fisheries and Aquaculture (IUPA), CheikhAnta Diop University (UCAD), Dakar, 5005, Senegal; b ANSES, Nancy Laboratory for Hydrology, Water Chemistry Department, 40 Rue Lionnois, Nancy, F-54000, France; c Université de Lorraine, INRAE, L2A, Nancy, F-54500, France; d Chambre d’Agriculture du Grand-Est, Laxou, F-54520, France

**Keywords:** Aquaculture, Environmental monitoring, Freshwater, Trace elements, Risk assessment, Water quality

## Abstract

Freshwater resources in Senegal play a crucial role in drinking water (DW) supply, agriculture, and the rapidly expanding aquaculture sector promoted under the National Sustainable Aquaculture Development Strategy. However, no nationwide assessment has previously investigated potentially toxic elements (PTEs) in freshwater resources supporting aquaculture. This study provides the first integrated evaluation of dissolved arsenic (As), cadmium (Cd), chromium (Cr), copper (Cu), nickel (Ni), lead (Pb), and zinc (Zn) in surface water and groundwater collected from 17 aquaculture farms distributed across the Senegal, Gambia, and Casamance river basins. Median PTE concentrations followed the order Cu > Zn > Ni > Cr > Pb ≈ As > Cd, and all measured concentrations remained below the corresponding World Health Organization DW guideline values. Human health risk assessment indicated that ingestion was the dominant exposure pathway, producing substantially higher chronic daily intake than dermal exposure for both adults and children. Nevertheless, hazard quotient, hazard index, and carcinogenic risk values remained below the United States Environmental Protection Agency acceptable thresholds, indicating no significant non-carcinogenic or carcinogenic health risks associated with the investigated PTEs. Principal component analysis identified distinct river-specific contamination signatures, with Zn, Cu, and Cd predominantly associated with the Casamance basin, whereas Cr and Pb were more strongly related to the Senegal River, while the Gambia River exhibited consistently low PTE concentrations. Overall, these findings indicate that freshwater resources supporting aquaculture in Senegal are currently of good chemical quality with respect to the investigated PTEs. This study provides the first national baseline for environmental monitoring and supports sustainable aquaculture development and water-resource management in Senegal.

## 1 Introduction

Freshwater pollution by potentially toxic elements (PTEs) has become a major global environmental and public health concern because of their persistence, toxicity, non-biodegradability, and tendency to bioaccumulate in aquatic ecosystems and food webs [1]. PTEs originate from both natural processes, including rock weathering and volcanic activity, and anthropogenic sources such as mining, industrial discharges, agricultural runoff, urbanization, and wastewater effluents [2]. Once introduced into freshwater environments, these contaminants can accumulate in sediments, aquatic organisms, and drinking water resources, posing serious ecological and human health risks through chronic exposure. Consequently, monitoring the occurrence, distribution, sources, and health implications of PTEs has become a global priority for sustainable water-resource management and environmental protection [3,4].

Across Africa, increasing population growth, rapid urbanization, agricultural intensification, mining activities, and climate change are placing unprecedented pressure on freshwater resources while environmental monitoring remains insufficient in many regions [5]. Rivers, lakes, and groundwater are essential sources of drinking water, irrigation, fisheries, and aquaculture, yet they are increasingly threatened by PTE contamination. Recent studies from Ghana, Ethiopia, Nigeria, and other African countries have reported elevated concentrations of arsenic (As), cadmium (Cd), chromium (Cr), copper (Cu), nickel (Ni), lead (Pb), and zinc (Zn), together with associated ecological and human health risks [3,4,6,7]. These investigations have demonstrated the importance of integrating contamination assessment, source identification, pollution indices, multivariate statistical analyses, and human health risk assessment to provide a comprehensive understanding of freshwater quality and to support evidence-based environmental management.

In Senegal, freshwater resources are increasingly important for domestic consumption, agriculture, livestock production, and the rapidly expanding aquaculture sector. Previous investigations have primarily focused on coastal ecosystems, estuaries, marine sediments, and localized mining areas [[8], [9], [10], [11], [12]]. These studies demonstrated that both anthropogenic activities and natural geological processes contribute to PTE occurrence in aquatic environments. However, most available information concerns sediments or coastal environments, whereas dissolved PTEs in inland freshwater systems remain poorly characterized. Consequently, knowledge regarding the occurrence, spatial distribution, and potential health implications of PTEs in freshwater resources supporting inland aquaculture is still limited.

This information gap is particularly important because Senegal has identified aquaculture as a strategic sector for improving food security, employment, and economic development through the National Sustainable Aquaculture Development Strategy [13]. The success of this strategy relies heavily on the availability of high-quality freshwater resources supplied by the Senegal, Gambia, and Casamance river systems, as well as associated groundwater. Although these waters constitute the primary source for fish farming activities, no comprehensive study has simultaneously investigated PTE occurrence, spatial distribution, pollution sources, and potential human health risks across the principal freshwater systems supporting aquaculture in Senegal. Furthermore, existing studies rarely integrate source identification techniques such as principal component analysis with quantitative human health risk assessment, limiting the understanding of contamination pathways and their implications for sustainable aquaculture development.

To address these knowledge gaps, the present study provides the first integrated assessment of PTEs (As, Cd, Cr, Cu, Ni, Pb, and Zn) in freshwater systems supporting aquaculture across the Senegal, Gambia, and Casamance river basins. Specifically, the objectives were (i) to determine the concentrations and spatial distribution of dissolved PTEs in surface water (SW) and groundwater (GW) used for aquaculture, (ii) to identify potential natural and anthropogenic sources, (iii) to evaluate non-carcinogenic and carcinogenic human health risks associated with ingestion and dermal exposure for adults and children following United States Environmental Protection Agency (USEPA) methodologies, and (iv) to provide baseline scientific evidence to support sustainable aquaculture development, environmental monitoring, and water resource management in Senegal.

## 2 Materials and methods

### 2.1 Study Area

The present study was conducted in Senegal (Figure S1), a West African country located between latitudes 12°18′ and 16°41′ N and longitudes 11°21′ and 17°32′ W, with a total area of approximately 196,700 km^2^. Senegal experiences a tropical climate characterized by two distinct seasons: a rainy season extending from June to October and a dry season from November to May. Mean annual precipitation ranges from less than 200 mm in the semi-arid northern regions to more than 1250 mm in the humid southern part of the country, while average annual temperatures generally vary between 24 and 30 °C. The country's hydrographic network is dominated by the Senegal, Gambia, and Casamance rivers, which constitute the principal freshwater resources supporting agriculture, domestic water supply, fisheries, livestock production, and the rapidly expanding aquaculture sector [14,15].

Consequently, maintaining good water quality is essential not only for fish health and productivity but also for safeguarding public health, as these water resources are frequently used simultaneously for domestic consumption, irrigation, livestock watering, and recreational purposes.

The Senegal River is the largest river basin in the country, extending over approximately 1800 km from the Fouta Djallon Highlands in Guinea through Mali and Mauritania before reaching the Atlantic Ocean at Saint-Louis. The river is regulated by the Manantali and Diama dams, which provide water for irrigation, hydropower generation, navigation, and aquaculture. Intensive agricultural activities, urban development, and localized industrial operations occurring within the basin represent potential sources of PTEs, particularly through agricultural runoff, domestic wastewater, and industrial effluents. The Gambia River originates in the Fouta Djallon massif of Guinea and flows through southeastern Senegal before entering The Gambia and discharging into the Atlantic Ocean. Compared with the Senegal River, this basin is less densely populated and experiences lower industrial activity. Nevertheless, artisanal gold mining, expanding agriculture, livestock production, and increasing aquaculture development constitute potential anthropogenic sources of PTEs. In addition, natural weathering of mineralized geological formations may contribute to the release of PTEsinto the aquatic environment. The Casamance River, located in southern Senegal, drains a humid tropical region characterized by abundant rainfall, mangrove ecosystems, floodplains, and extensive agricultural activities. The river supports numerous freshwater aquaculture farms owing to its relatively stable hydrological conditions throughout much of the year. However, agricultural runoff, urban expansion, navigation, domestic wastewater discharges, and natural geochemical processes may influence water quality and increase the concentrations of dissolved PTEs within the basin. These three river systems were selected because they represent the principal freshwater environments currently supporting inland aquaculture development in Senegal while encompassing contrasting climatic, geological, hydrological, and socio-economic conditions.

From a public health perspective, assessing PTE contamination in these freshwater systems is particularly important because local communities frequently rely on these waters for drinking, food preparation, bathing, irrigation, and fish production.

Sampling activities were conducted with the collaboration and logistical support of the National Aquaculture Agency (ANA) within the framework of the national aquaculture development program. Access to aquaculture facilities was granted by farm owners and local authorities prior to sampling.

### 2.2 Sampling strategy and analysis

A cross-sectional analytical study was conducted between June and August 2022 to characterize the occurrence, spatial distribution, and potential sources of dissolved PTEs in freshwater resources supporting aquaculture production in Senegal. A cross-sectional design was selected because it provides a representative assessment of water quality during the principal aquaculture production period while allowing comparison among geographically distinct river systems under similar hydrological conditions. This approach has been widely adopted in environmental monitoring studies as a baseline framework for contamination profiling, source identification, and probabilistic human health risk assessment [3,4].

The sampling campaign targeted freshwater resources used directly for aquaculture production, including both SW and GW. Including both water sources was considered essential because Senegalese aquaculture systems rely on rivers, reservoirs, wells, and boreholes depending on seasonal water availability. During the dry season, GW frequently becomes the principal source of water for fish farming, whereas SW predominates during periods of higher discharge. Consequently, evaluating both sources provide a more comprehensive assessment of potential human and environmental exposure to PTEs.

Sampling locations were selected from the national inventory of commercial aquaculture farms established during the implementation of the SNDAq [13]. Site selection was based on four principal criteria: (i) active aquaculture production at the time of sampling, (ii) dependence on freshwater resources for pond supply, (iii) geographical representation of the three principal river basins supporting aquaculture development, and (iv) logistical accessibility during the sampling campaign. Seventeen farms fulfilled these criteria and were retained for the study. Because one farm utilized both SW and GW (river and borehole), a total of eighteen water samples were collected.

The sampling network included seven farms located within the Senegal River basin (Farms 1–7), two farms supplied by the Gambia River (Farms 8 and 9), and eight farms distributed throughout the Casamance River basin (Farms 10–17). This spatial distribution ensured broad geographical coverage across Senegal while encompassing diverse climatic, geological, hydrological, and land-use conditions. The geographic coordinates for each sampling location, farm names, and corresponding water sources used for fish farming are listed in Table 1.

**Table 1:** Description of the aquaculture farms, geographical coordinates, and freshwater sources included in the study.

Farm Number	Farm Name	Geographical Coordinates	Area	Water sources
F1–1	ANA	15°38′29.1″N - 13°14′49.6″W	Matam	SW (Senegal river)
F1–2	ANA	15°38′29.1″N - 13°14′49.6″W	Matam	GW (Senegal basin)
F2	Ndiadjé	15°25′16.9″N - 13°06′10.0″W	Matam	SW (Senegal river)
F3	Yague	16°27′47.5″N - 15°41′36.4″W	Richard-Toll	SW (Senegal river)
F4	Fall	16°28′07.0″N - 15°42′01.0″W	Richard-Toll	SW (Senegal river)
F5	Talla	16°28′09.5″N - 15°42′15.9″W	Richard-Toll	SW (Senegal river)
F6	OLAC	16°03′49.0″N - 16°24′42.8″W	Gandon	SW (Senegal river)
F7	UGB	16°03′22.2″N - 16°25′40.7″W	Gandon	SW (Senegal river)
F8	DAC/Itato	12°30′24.0″N - 12°13′52.0″W	Bandafassi	SW (Gambia river)
F9	Ba	12°36′09.4″N - 12°07′23.0″W	Bandafassi	SW (Gambia river)
F10	Sow-ranch	12°54′46.8″N - 14°55′15.7″W	Dioulacolon	GW (Casamance basin)
F11	Maître	12°54′39.7″N - 14°55′15.4″W	Dioulacolon	GW (Casamance basin)
F12	Coly	12°50′12.9″N - 15°56′43.9″W	Marsassoum	GW (Casamance basin)
F13	DAC/Sefa	12°51′08.3″N - 15°37′22.2″W	Diendé/ Koussi	GW (Casamance basin)
F14	Agnak	12°33′59.0″N - 16°06′43.8″W	Niaguis	GW (Casamance basin)
F15	Sœurs	12°33′49.4″N - 16°12′50.5″W	Niaguis	GW (Casamance basin)
F16	Samuel	12°31′30.0″N - 16°14′58.4″W	Niaguis	GW (Casamance basin)
F17	Goudiaby	12°35′03.8″N - 16°17′53.8″W	Ziguinchor	SW (Casamance river)

Surface water: SW; Groundwater: GW

Sampling sites were selected to represent different sections of the rivers whenever possible. Upstream locations were chosen to characterize areas experiencing relatively limited anthropogenic influence and to provide reference conditions representative of background water quality. Midstream sites were selected in zones influenced by agricultural production, rural settlements, and aquaculture activities, where cumulative watershed inputs were expected to increase. Downstream sites represented areas potentially affected by integrated upstream discharges, urban activities, irrigation return flows, and estuarine processes.

SW samples were taken from the outlet of the supply pipe delivering river water to the aquaculture ponds. Before collection, the pumping system was operated for several minutes to flush stagnant water and ensure that the sample reflected freshly pumped river water. GW samples were collected from the borehole or well outlet supplying the ponds, after similarly flushing the system for several minutes to obtain representative sample. In both cases, water was collected directly into pre-cleaned 1 L glass bottles conforming to ISO 4796–1:2016 standards. At each location, sampling containers were rinsed three times with ambient water before final collection to reduce the possibility of cross-contamination.

All sampling equipment was thoroughly cleaned before field deployment using laboratory detergent, rinsed with ultrapure water, soaked for at least 24 h in 10% nitric acid (HNO_3_), and finally rinsed several times with ultrapure water. This cleaning protocol minimized the risk of contamination during sampling and transportation.

Immediately after collection, water samples were filtered through pre-cleaned 0.45 μm glass microfiber (GF/A) filters to obtain the dissolved fraction of PTEs. Filtered samples were transferred into acid-washed polypropylene Falcon tubes and stored in insulated coolers containing ice packs throughout transportation to the laboratory. Within 24 h of collection, samples were preserved by acidification with ultrapure HNO_3_ to a final concentration of approximately 3% (v/v), thereby preventing PTE precipitation, adsorption onto container walls, and microbial alteration prior to chemical analysis. Samples were subsequently stored at −18 °C until laboratory analysis. Concentrations of Cd, Cu, Pb, Ni, Cr, Zn, and As in the dissolved phase were analyzed by inductively coupled plasma mass spectrometry (ICP-MS) at the *Centre de Recherches Pétrographiques et Géochimiques* (CRPG, Nancy, France). The limits of quantification (LOQ) were 0.005, 0.002, 0.01, 0.01, 0.04, 0.015, and 0.025 μg L^−1^ for Cd, Cu, Pb, Ni, Cr, Zn, and As, respectively.

To ensure sample integrity throughout the sampling campaign, each container was assigned a unique identification code indicating the river basin, farm number, sampling date, and water source. Samples were transported under refrigerated conditions and handled according to internationally accepted protocols for trace element analysis to minimize contamination and preserve analytical quality.

### 2.3 Human health risk assessment (HHRA)

The potential human health implications associated with exposure to PTEs were evaluated using the United States Environmental Protection Agency (USEPA) human health risk assessment (HHRA) methodology. HHRA provides a quantitative framework for estimating both non-carcinogenic and carcinogenic risks resulting from long-term exposure to environmental contaminants and has been widely applied in water quality assessments to identify priority contaminants, vulnerable populations, and potential public health concerns [3,4,6,7].

The assessment considered two primary exposure pathways: ingestion, representing the direct consumption of contaminated water, and dermal absorption, representing skin contact during activities such as bathing, swimming, washing, and aquaculture-related practices. The dermal pathway was included because direct contact with river water is common among communities living along the Senegal and Gambia rivers [[16], [17], [18]]. In contrast, the inhalation pathway was excluded because dissolved PTEs exhibit negligible volatility and limited potential for aerosolization under normal environmental conditions.

Human health risks were assessed separately for adults and children by calculating the chronic daily intake (CDI), hazard quotient (HQ), hazard index (HI), and carcinogenic risk (CR) for each exposure pathway. All exposure assumptions, toxicity parameters, and calculation procedures were selected according to standard USEPA guidelines and the published literature to ensure consistency with internationally accepted HHRA methodologies.

#### 2.3.1 Chronic daily intake (CDI)

The CDI (mg kg⁻^1^ day⁻^1^) of each PTE was calculated for both ingestion and dermal exposure using (1), (2) respectively [19], [20], [21].

(1) $${\mathrm{CDI}}_{\mathrm{ingestion}}=\frac{C_{w}{\times \mathrm{IR}}_{w}\times \mathrm{EF}\times \mathrm{ED}}{\mathrm{BW}\times \mathrm{AT}}$$

(2) $${\mathrm{CDI}}_{\mathrm{dermal}}=\frac{C_{w}\times \mathrm{SA}\times K_{p}\times \mathrm{ET}\times \mathrm{EF}\times \mathrm{ED}\times \mathrm{ABS}}{\mathrm{BW}\times \mathrm{AT}}$$

where *Ci* represents the concentration of PTE in water (mg L⁻^1^); *EF* is the exposure frequency (days year⁻^1^); *IRw* denotes the daily water ingestion rate (L day⁻^1^); *ED* is the exposure duration (years); *BW* corresponds to the body weight (kg); and *AT* is the averaging time (days) for non-carcinogenic or cancerogenic exposure. *SA* refers to the skin surface area in contact with water (cm^2^); *Kp* is the dermal permeability coefficient (cm h⁻^1^); *ET* is the daily exposure time (h day⁻^1^); and *ABS* represents the dermal absorption fraction. The parameter values applied in the estimation of CDI through ingestion and dermal contact are listed in Table 2, while the specific *Kp* values for As, Cd, and Cu is 0.001 cm h⁻^1^, for Ni and Pb is 0.004 cm h⁻^1^, and those for Cr and Zn are respectively 0.002 and 0.006 cm h⁻^1^ [22,23,24].

**Table 2:** Exposure parameters for child and adult used for CDI calculations.

Parameter	Adult	Child	Unit
IRw (ingestion rate)	2.5	1.0	L day⁻^1^
EF (exposure frequency)	350	350	days year⁻^1^
ED (exposure duration)	30	6	years
BW (body weight)	70	15	kg
AT (averaging time, non-carcinogenic)	10950	2190	days
AT (averaging time, carcinogenic)	25550	25550	days
SA (skin surface area exposed)	18000	6600	cm^2^
ET (exposure time)	0.58	1.00	h day⁻^1^
ABS (dermal absorption fraction)	0.001	0.001	L cm⁻^3^

#### 2.3.2 Non-carcinogenic risk

The hazard quotient (HQ) was applied to evaluate potential non-carcinogenic health risks arising from human exposure to PTE in water. In this assessment, both major exposure pathways related to water use or consumption (i.e., dermal contact and ingestion) were considered when calculating HQ values ((3), (4)) [25,26]. If HQ is less than one, then the condition is considered not hazardous and safe to human health [26,27].

(3) $${\mathrm{HQ}}_{\mathrm{ingestion}}=\frac{{\mathrm{CDI}}_{\mathrm{ingestion}}}{{\mathrm{RfD}}_{\mathrm{ingestion}}}$$

(4) $${\mathrm{HQ}}_{\mathrm{dermal}}=\frac{{\mathrm{CDI}}_{\mathrm{dermal}}}{{\mathrm{RfD}}_{\mathrm{dermal}}}$$

where CDI represents the chronic daily intake, and RfD (mg kg⁻^1^ day⁻^1^) is the reference dose, defined as the daily exposure level considered safe over a lifetime (see Table 3).

**Table 3:** Reference dose for non-carcinogenic PTE (mg kg⁻^1^ day⁻^1^) and slope factor for carcinogenic PTE (mg kg⁻^1^ day⁻^1^)^−1^ for ingestion and dermal [[28], [29], [30], [31]].

	RfD_ing_	RfD_derm_	SF_ing_	SF_derm_
Zn	3.00e-01	6.00e-02	-	-
Cu	3.70e-02	1.10e-02	-	-
Ni	2.00e-02	1.00e-03	1.70e + 00	2.00e + 01
Cr	3.00e-03	2.50e-04	5.00e-01	2.00e + 01
As	3.00e-04	2.70e-04	1.50e + 00	3.66e + 00
Pb	3.50e-03	5.30e-04	8.50e-03	1.50e + 00
Cd	1.00e-03	5.00e-05	1.50e + 01	6.30e + 00

The hazard index (HI) was employed to evaluate the overall non-carcinogenic health risk resulting from exposure to multiple contaminants. It is calculated as the sum of the individual HQ. In this study, both relevant exposure pathways associated with the consumption and use of water were included in the HI calculation. The applied equations are shown in (5), (6), (7) [26].

(5) $${\mathrm{HI}}_{\mathrm{ingestion}}=\sum_{k=1}^{n}\mathrm{HQ}=\;\sum {\mathrm{HQ}}_{\mathrm{As}}+{\mathrm{HQ}}_{\mathrm{Cd}}+{\mathrm{HQ}}_{\mathrm{Cr}}+{\mathrm{HQ}}_{\mathrm{Cu}}+{\mathrm{HQ}}_{\mathrm{Ni}}+{\mathrm{HQ}}_{\mathrm{Pb}}+{\mathrm{HQ}}_{\mathrm{Zn}}$$

(6) $${\mathrm{HI}}_{\mathrm{dermal}}=\sum_{k=1}^{n}\mathrm{HQ}=\;\sum {\mathrm{HQ}}_{\mathrm{As}}+{\mathrm{HQ}}_{\mathrm{Cd}}+{\mathrm{HQ}}_{\mathrm{Cr}}+{\mathrm{HQ}}_{\mathrm{Cu}}+{\mathrm{HQ}}_{\mathrm{Ni}}+{\mathrm{HQ}}_{\mathrm{Pb}}+{\mathrm{HQ}}_{\mathrm{Zn}}$$

where HI is the hazard index for ingestion and dermal, while HQ is the hazard quotient for the corresponding PTE.

(7) $$\mathrm{THI}=\;{\mathrm{HI}}_{\mathrm{ingestion}}+\;{\mathrm{HI}}_{\mathrm{dermal}}$$

where THI is the total hazard index for both exposure route.

#### 2.3.3 Carcinogenic risk (CR)

CR represents the estimated probability of developing cancer over a lifetime as a result of exposure to carcinogenic substances in water. In this study, both exposure routes linked to water use and consumption were incorporated into the CR assessment. The corresponding equations are estimated using (8), (9), (10).

(8) $${\mathrm{CR}}_{\mathrm{ingestion}}=\;{\mathrm{CDI}}_{\mathrm{ingestion}}\;\times \;{\mathrm{CSF}}_{\mathrm{ingestion}}$$

(9) $${\mathrm{CR}}_{\mathrm{dermal}}=\;{\mathrm{CDI}}_{\mathrm{dermal}}\;\times \;{\mathrm{CSF}}_{\mathrm{dermal}}$$

where CR is the carcinogenic risk, CDI is the chronic daily intake, and CSF is the cancer slope factor ((mg kg⁻^1^ day⁻^1^)⁻^1^) (see Table 3).

(10) $$\mathrm{TCR}=\;{\mathrm{CR}}_{\mathrm{ingestion}}+\;{\mathrm{CR}}_{\mathrm{dermal}}$$

where TCR is the total cancer risk.

The USEPA defines CR values lower than 1e−06 as negligible, between 1e−06 and 1e−04 as acceptable, and above 1e−04 as potentially significant [32].

### 2.4 Statistical analysis

Descriptive statistical analyses were performed to characterize the distribution of PTE concentrations in aquaculture waters. For each element, the median, minimum, maximum, first quartile (Q1), third quartile (Q3), and interquartile range (IQR) were calculated. Because concentrations exhibited right-skewed distributions and spanned several orders of magnitude, results were summarized using medians and quartiles and visualized on logarithmic (log_10_) scales where appropriate.

Principal component analysis (PCA) was conducted to explore multivariate relationships among PTEs and to identify spatial patterns across the Senegal, Gambia, and Casamance river basins. Prior to PCA, concentration data were standardized using z-score normalization (mean = 0, standard deviation = 1) to eliminate differences in measurement scales among PTEs. PCA was performed using the correlation matrix, and the first two principal components were retained for interpretation. Loading vectors were used to identify the PTEs contributing most strongly to each principal component, while sampling sites were projected onto the PCA space to visualize similarities and basin-specific clustering.

All statistical analyses, calculations, and graphical visualizations were performed using R (version 4.4.1) within the RStudio environment.

### 2.5 Quality assurance / quality control

Quality assurance and quality control procedures were implemented throughout sample preparation and instrumental analysis to ensure the accuracy, precision, and reliability of the analytical results. Calibration curves were prepared using multi-element standard solutions (Agilent Technologies, USA; Ref. Nos. 8500–6940, 8500–6942, and 8500–6948) at concentrations ranging from 0.05 to 500 µg L⁻^1^. Five calibration points were generated by serial dilution with ultrapure water, and only calibration curves with correlation coefficients (R² > 0.99) were accepted for quantification.

Instrument performance was optimized before each analytical batch using a 1 µg L⁻^1^ multi-element tuning solution (Agilent Technologies, USA) containing a broad mass range of elements. Analytical precision was evaluated through duplicate analyses and repeated measurements, yielding relative standard deviations (RSD) below 5%. Method accuracy was verified using the certified reference material NIST SRM 1643 f (PTEs in Water), with recoveries ranging from 90% to 110%.

Quality control during analysis included procedural blanks, instrument blanks, field blanks, and replicate samples to monitor potential contamination and analytical variability. Blank corrections were applied where appropriate, and no detectable contamination was observed in any of the field blanks. All water samples were analyzed in triplicate to ensure analytical reproducibility.

## 3 Results and discussion

### 3.1 PTE concentration

The summary of PTE concentrations (Fig. 1) highlighted significant contrasts in both magnitude and variability among the analyzed elements. Median concentrations followed the decreasing order Cu > Zn > Ni > Cr > Pb ~ As > Cd. indicating that Cu (1.94 µg L⁻^1^) and Zn (1.67 µg L⁻^1^) were the dominant PTEs in the Senegal aquaculture waters. Both Zn and Cu displayed wide concentration ranges, particularly in one sample (F16) collected from Casamance River, with maxima of 52.45 µg L⁻^1^ and 12.71 µg L⁻^1^, respectively. These elevated upper values, combined with large interquartile ranges (Q1–Q3), reflected substantial spatial heterogeneity, most likely reflect combined lithogenic contributions from mineral weathering [33] and localized anthropogenic inputs, including metal release from corroded metal structures [34], runoff from metal roofing [35] and urban areas [36], and agricultural runoff [37,38]. Nickel (median of 0.62 µg L⁻^1^) and chromium (median of 0.38 µg L⁻^1^) displayed moderate variability, with upper quartiles around 1.00 µg L⁻^1^ and 0.54 µg L⁻^1^, respectively. This pattern pointed to mixed lithogenic and anthropogenic sources. Natural mineral weathering or groundwater-rock interactions may contribute to background levels [33], which is consistent with the presence of Ni and Cr formations documented in West Africa [39], while local human activities could occasionally enhance concentrations. As concentrations ranged from 0.05 to 1.80 µg L⁻^1^, showing a moderately skewed distribution suggestive of geogenic origin, possibly influenced by sediment redox processes typical of tropical aquifer systems [40]. In contrast, Pb and Cd showed narrow ranges (Pb: 0.14–0.44 µg L⁻^1^; Cd: 0.01–0.03 µg L⁻^1^) with very low dispersion, indicating uniformly low background concentrations and limited anthropogenic impact. Their medians (Pb 0.25 µg L⁻^1^; Cd 0.01 µg L⁻^1^) were well below typical guideline thresholds for SW used in aquaculture, suggesting negligible contamination risk for these two elements. Overall, the data indicated that Cu, Zn, and Ni were the most variable and potentially site-influenced PTEs, whereas Pb and Cd remained consistently low. The relatively moderate levels observed for all PTEs were within or below international guideline limits [41] implying that there was no widespread PTE pollution in Senegalese aquaculture waters. However, the occurrence of isolated high Zn and Cu concentrations highlighted the need for targeted monitoring to prevent potential bioaccumulation risks in farmed fish and sediments.

**Fig. 1:**
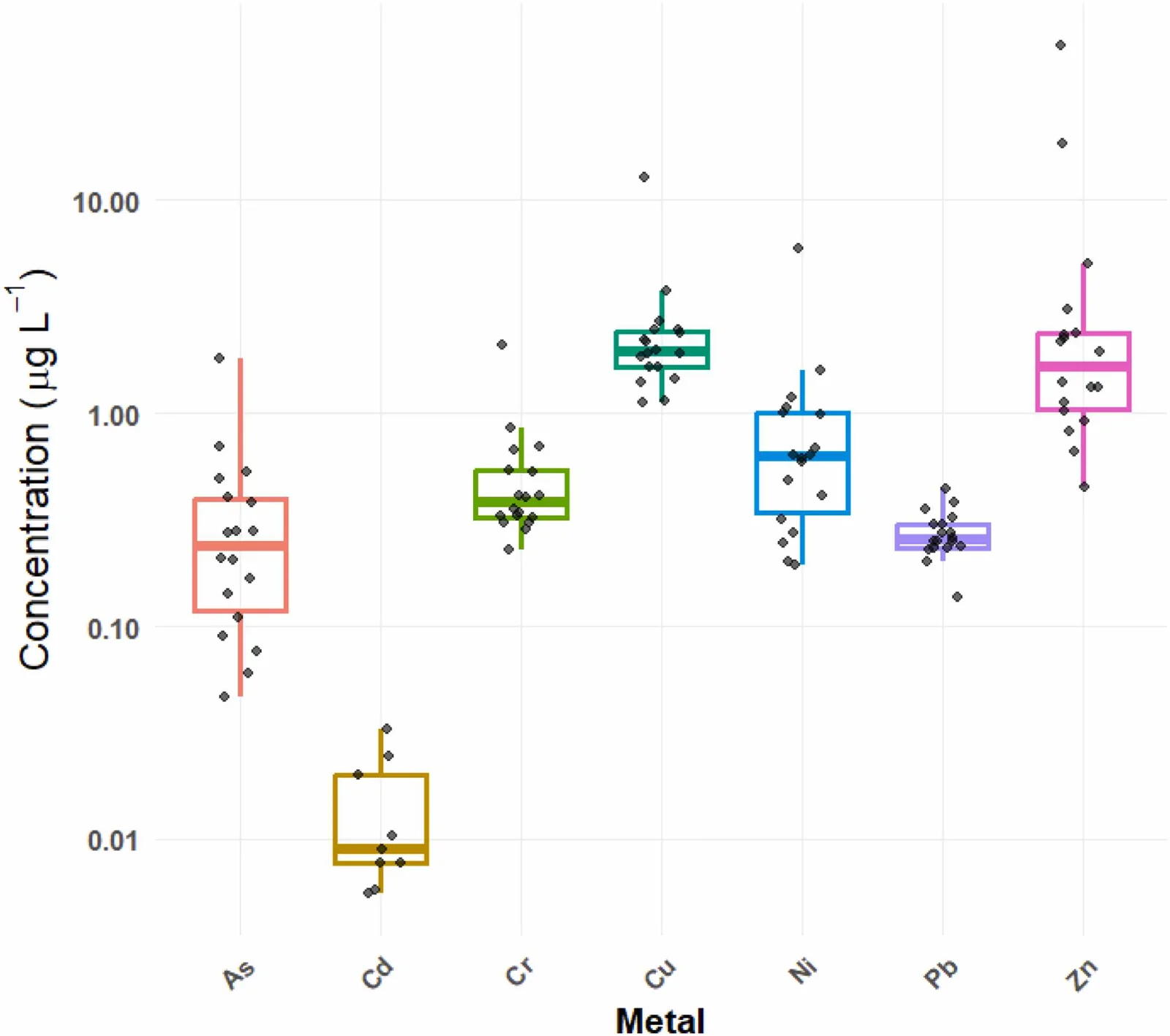
Dissolved PTE concentrations (µg L⁻^1^) in Senegal water samples. Values are presented on a logarithmic (log_10_) scale.

The spatial distribution of the investigated PTEs across the Senegal, Gambia, and Casamance rivers is illustrated in Fig. 2. In the Senegal River, median concentrations of most PTEs were slightly higher upstream than downstream, showed minimal variation, indicating overall low spatial heterogeneity. In contrast, the Gambia River displayed a different pattern, with slightly higher concentrations downstream for certain elements, suggesting localized enrichment in the lower reaches. Upstream-downstream increases were evident for Cu (1.40–2.44 µg L⁻^1^), Zn (0.45–2.16 µg L⁻^1^), and Ni (0.27–0.68 µg L⁻^1^). The geological basement of eastern Senegal belongs to the Paleoproterozoic Birimian formations [42]. This unit, known to host copper and zinc [43], may progressively release these elements through meteoric weathering along the upstream–downstream gradient of the Gambia River. This trend may also reflect the downstream transfer of fine particles derived from artisanal gold-mining areas in the upper basin, a process known to enhance PTE loads in rivers draining the Kédougou region [11]. An upstream-downstream contrast occurred in the Casamance River, where median concentrations of Zn and Cr increased markedly from 2.02 to 3.68 µg L⁻^1^ and from 0.38 to 0.54 µg L⁻^1^, respectively. Cu remained high throughout (2.18–2.22 µg L⁻^1^), while As and Ni showed moderate reductions downstream (0.09–0.12 µg L⁻^1^ and 1.08–0.62 µg L⁻^1^, respectively). These downstream enrichments, particularly for Zn and Cu, point to cumulative anthropogenic influence, likely arise from multiple anthropogenic sources. Possible contributions include lithogenic release from tropical lateritic soils under seasonal redox changes [40], dissolution of PTEs from corroded metallic infrastructures such as pumps or galvanized surfaces [35], diffuse inputs from populated areas with untreated effluents [36], and agricultural runoff linked to fertilizer and organic amendments [37]. Given this combination of processes, it remains difficult to attribute the observed downstream increases to a single dominant driver.

**Fig. 2:**
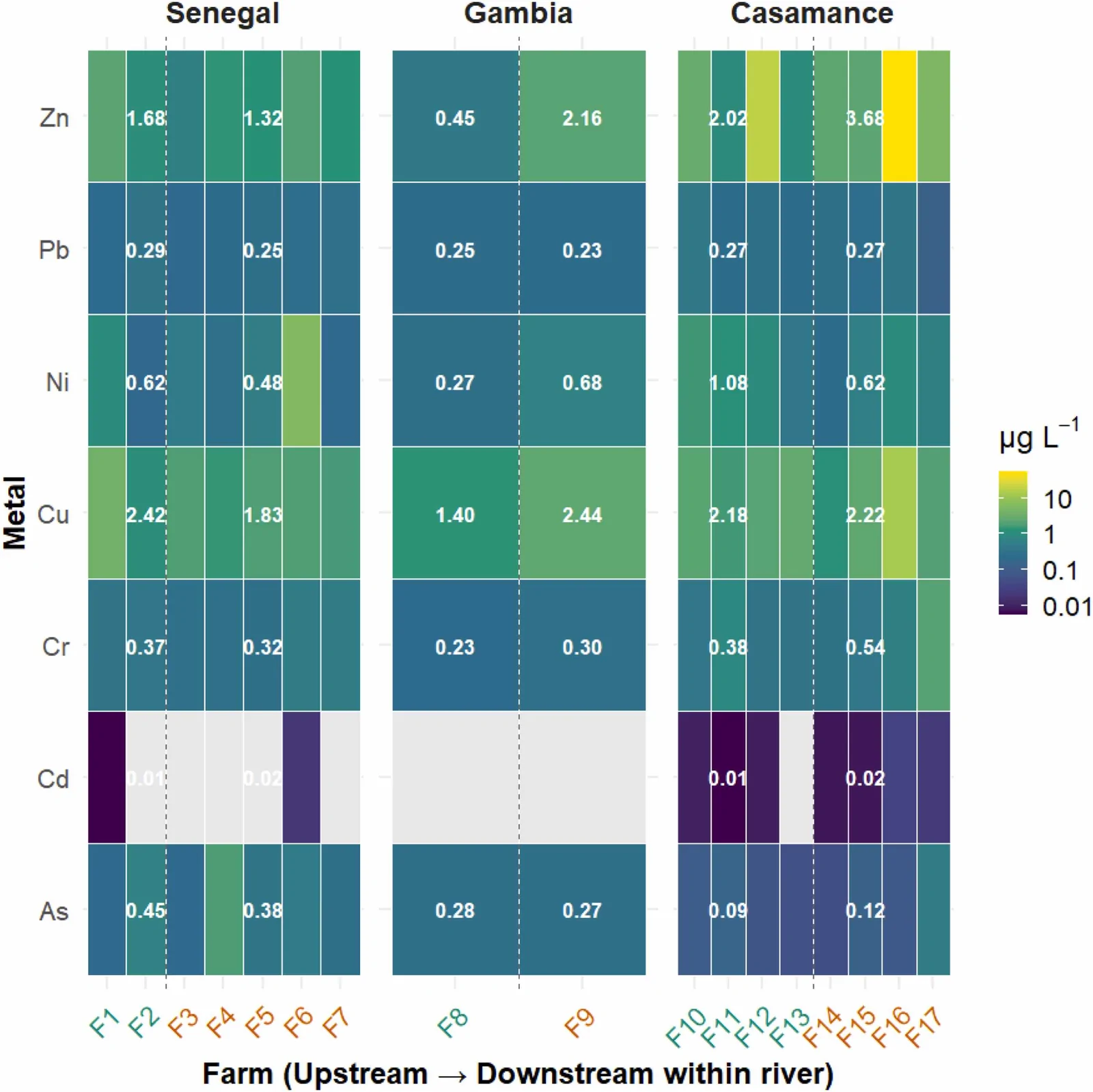
Heatmap of PTE concentrations (µg L⁻^1^) in aquaculture farms located along the Senegal, Gambia, and Casamance rivers. Sites (on the x-axis) are arranged from upstream (green labels) to downstream (orange labels). Values within boxes represent median concentrations for upstream and downstream sites in each river.

### 3.2 Human health risk assessment

#### 3.2.1 Chronic daily intake (CDI)

The estimated CDI values revealed that ingestion was the dominant exposure pathway for all investigated PTEs, exceeding dermal exposure by approximately two to four orders of magnitude in both adults and children (Fig. 3). This pattern is expected because the gastrointestinal tract absorbs dissolved PTEs much more efficiently than intact human skin, whose low permeability and modest dermal absorption coefficients substantially limit transdermal uptake [44]. Similar observations have been reported in freshwater risk assessments from Africa and elsewhere, where ingestion consistently represents the principal contributor to human exposure [29,45]. Consequently, dermal exposure associated with routine activities such as bathing, washing, swimming, and aquaculture-related work is expected to represent only a secondary exposure pathway.

**Fig. 3:**
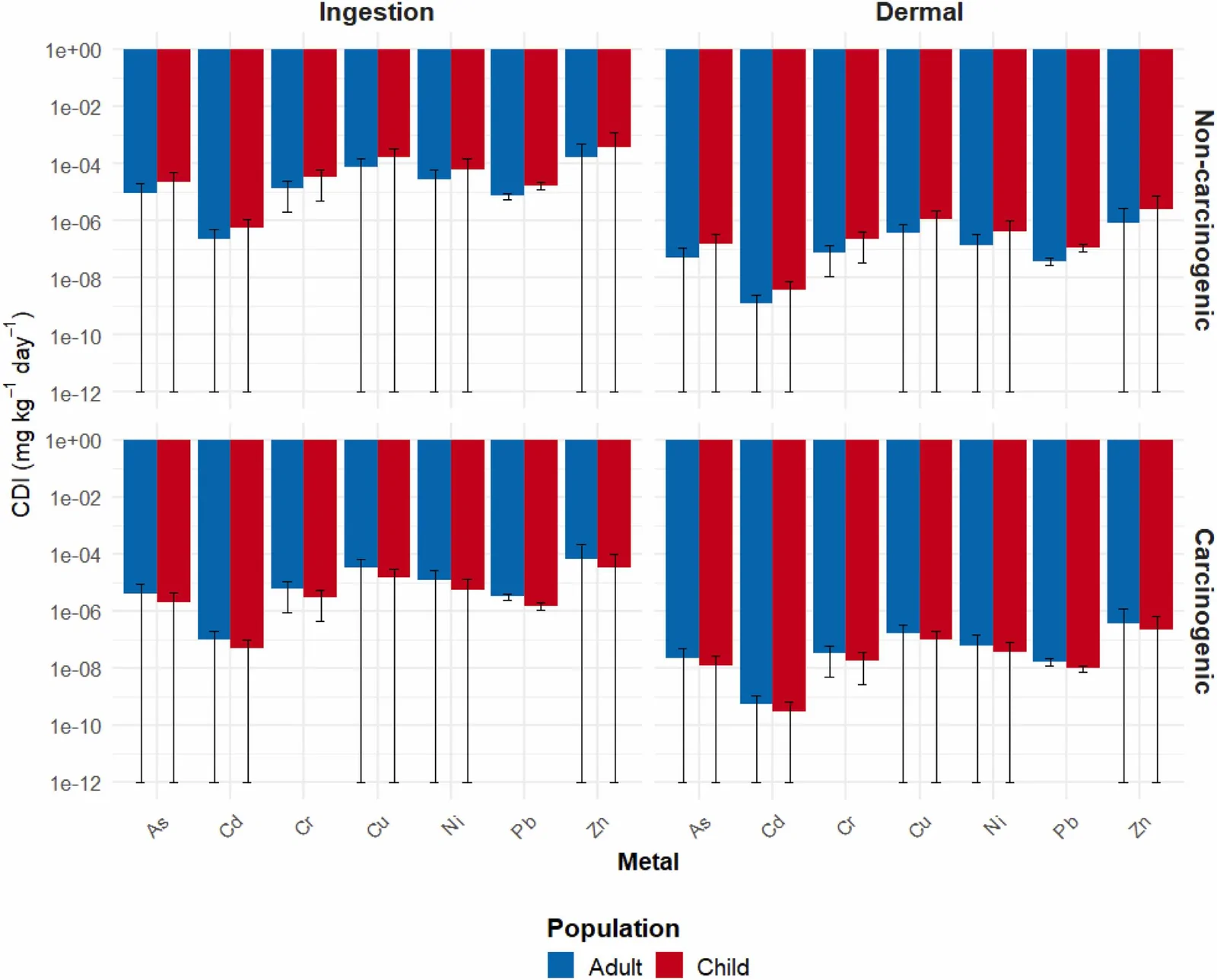
Mean ± SD of chronic daily intake (CDI, mg kg⁻^1^ day⁻^1^) for adults and children via ingestion and dermal exposure, categorized by carcinogenic and non-carcinogenic pathways. Values are presented on a logarithmic (log_10_) scale.

Among the investigated PTEs, Zn and Cu produced the highest CDI values owing to their comparatively higher dissolved concentrations in aquaculture waters, whereas Cd consistently exhibited the lowest exposure levels. Although CDI values varied among the three river basins, these differences primarily reflected the spatial distribution of PTE concentrations rather than differences in exposure pathways. In particular, the relatively elevated Zn and Cu concentrations measured in the Casamance basin resulted in higher estimated intake compared with the Senegal and Gambia river systems. Children generally exhibited higher body-weight-normalized CDI values than adults, with a child-to-adult ratio of approximately 2.3, owing to their lower body mass, greater water intake relative to body weight, and their increased hand-to-mouth activity, which enhances incidental ingestion [46]. Nevertheless, all estimated CDI values remained low and primarily served as input parameters for subsequent non-carcinogenic and carcinogenic risk assessment rather than indicating adverse health effects.

#### 3.2.2 Hazard quotient (HQ) and hazard index (HI)

The calculated HQ and cumulative HI demonstrated that exposure to the investigated PTEs through both ingestion and dermal contact did not pose significant non-carcinogenic health risks (Fig. 4). For both adults and children, all individual HQ values and cumulative HI values remained well below the USEPA safety threshold of 1, indicating that long-term exposure to the investigated freshwater resources is unlikely to produce adverse non-cancer health effects under the assessed exposure scenarios.

**Fig. 4:**
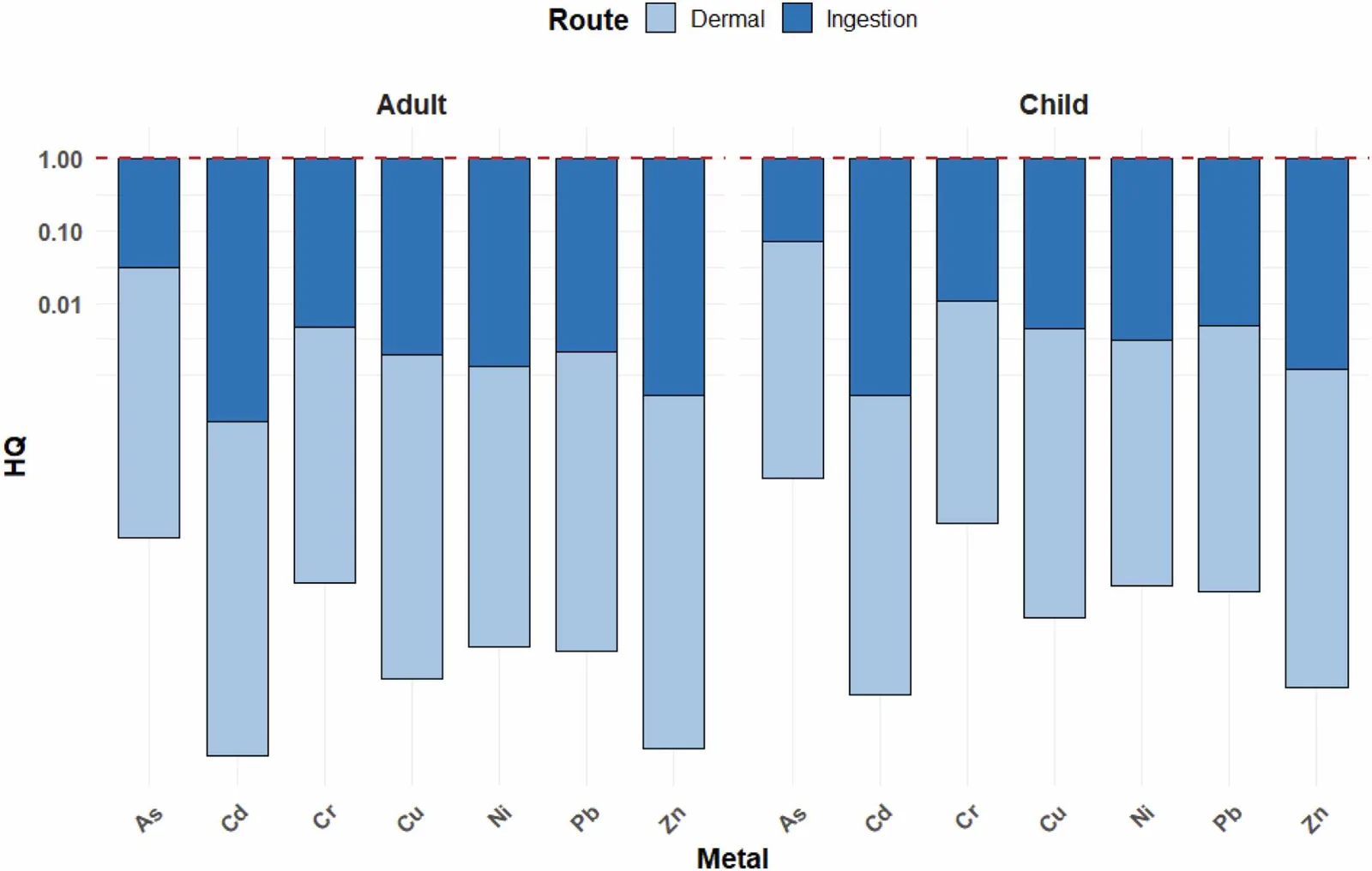
HQ via ingestion and dermal exposure routes for adults and children across studied PTEs. The dashed red line represents the safety threshold (HQ = 1). Values are presented on a logarithmic (log_10_) scale.

Among the investigated PTEs, As and Cr were the principal contributors to the cumulative HI, accounting for approximately 25% and 40%, respectively, of the overall non-carcinogenic risk. Consequently, the overall trend in mean HI values (ingestion + dermal) for both adults and children followed the order As > Cr > Pb > Cu > Ni > Zn > Cd. Despite their relatively low environmental concentrations, As and Cr exerted the greatest influence on the cumulative HI because of their comparatively low toxicological reference doses, which increase their relative contribution to risk estimates. These observations emphasize that non-carcinogenic risk depends not only on the environmental concentration of individual PTEs but also on their intrinsic toxicological characteristics and absorbed dose [2]. Similar findings have been reported in freshwater systems where As and Cr dominated cumulative non-carcinogenic risk despite concentrations remaining below DW guideline values [4,6,30,47]. The relatively greater contribution of both PTEs (As and Cr) suggests that they warrant continued attention in future monitoring and risk management programs. This is particularly relevant for As, which ranks first on the Agency for Toxic Substances and Disease Registry (ATSDR) 2022 Substance Priority List because of its toxicity, frequency of occurrence, and potential for human exposure [48].

Children consistently exhibited higher HQ and HI values than adults. Nevertheless, the cumulative HI remained below unity for both age groups, indicating that neither adults nor children are expected to experience adverse non-carcinogenic health effects under current exposure conditions. Similar child-dominated exposure patterns have been widely documented in drinking-water risk assessments worldwide [28,[49], [50], [51]].

Consistent with the CDI results, ingestion represented the principal contributor to non-carcinogenic risk, whereas dermal exposure accounted for only a negligible fraction of the total HI. Similar studies have reported that dermal exposure contributes less than 1% of the total non-carcinogenic risk in freshwater systems [30,52]. Overall, these findings indicate that freshwater resources supporting aquaculture in the Senegal, Gambia, and Casamance river basins currently present low non-carcinogenic health risks for both adults and children.

#### 3.2.3 Carcinogenic risk (CR)

The estimated CR associated with As, Cd, Cr, Ni, and Pb remained within the acceptable risk range (10^−6^–10^−4^) recommended by the USEPA for both adults and children (Fig. 5). Among the investigated PTEs, Ni contributed the greatest proportion of the cumulative CR, followed by Cr and As. However, all calculated CR values remained within the USEPA acceptable risk range, indicating that the investigated waters are unlikely to pose significant CRs under current exposure conditions. Similar findings have been reported in recent studies, where Ni emerged as the principal contributor to CR despite overall risks remaining within acceptable limits [30,[49], [50], [51],^53^]. Adults exhibited slightly higher cumulative CR values than children, reflecting the exposure assumptions adopted in the USEPA lifetime risk model and the longer cumulative duration of exposure through bathing, washing, and household activities. Dermal exposure represented only a small proportion of the total CR, contributing approximately 6% of the total CR for most PTEs, while ingestion accounted for more than 80% of the cumulative risk in both adults and children. Overall, the HHRA indicates that freshwater resources supporting aquaculture in the investigated river basins currently pose a low carcinogenic risk to exposed populations. Nevertheless, continued environmental monitoring remains essential because increasing agricultural activities, urbanization, and aquaculture development may alter future PTE inputs and associated health risks.

**Fig. 5:**
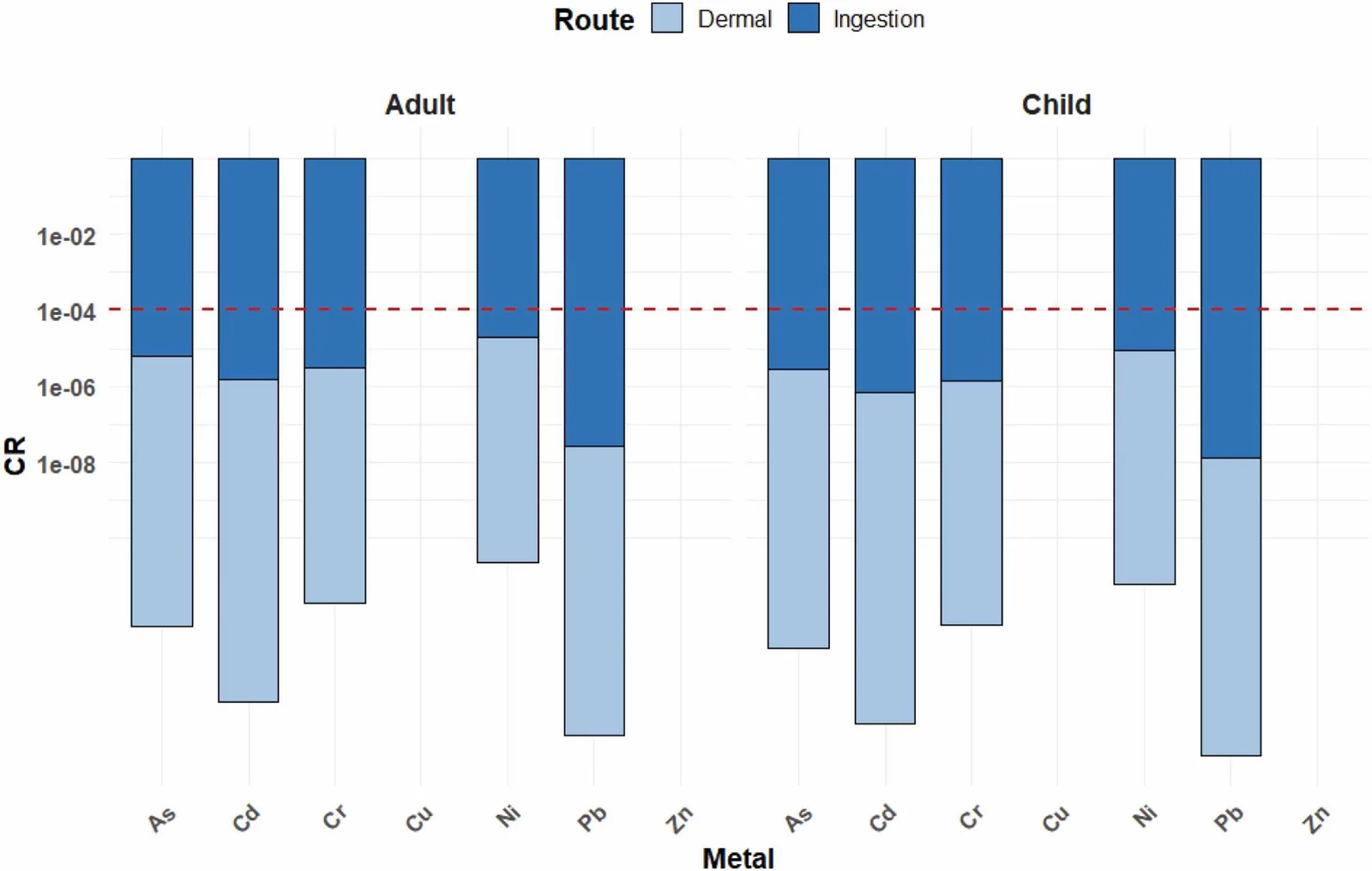
CR via ingestion and dermal exposure routes for adults and children across studied PTEs. The red dashed line denotes the target risk level (1e−04). Values are presented on a logarithmic (log_10_) scale.

### 3.3 Principal component analysis (PCA)

The principal component analysis (PCA) revealed clear spatial structuring of PTE contamination across the three river systems, with the first two components explaining 64% of the total variance (PC1 = 40.9%, PC2 = 22.9%) (Fig. 6). PC1 represented a major contamination gradient strongly influenced by Zn, Cu, and Cd, which exhibited high negative loadings (–0.551, –0.540, and –0.521, respectively) and together explained more than 86% of the variance within this axis. The coherent orientation of these loadings suggests a tightly correlated Zn–Cu–Cd assemblage that likely arises from common anthropogenic pressures, such as agricultural practices or stormwater runoff from metal roofs [35]. Farms located in the Casamance basin projected strongly along the negative side of PC1, highlighting their relatively elevated concentrations of these PTEs and forming a clearly identifiable cluster. PC2 captured a second and more complex gradient dominated by Cr and Pb, which displayed contrasting loadings; Cr with a strong negative loading (–0.648, 42% contribution) and Pb with a strong positive loading (0.570, 32% contribution). This opposition suggests the influence of two distinct geochemical or contamination processes. Cr enrichment may reflect lithogenic sources or sediment resuspension, whereas Pb enrichment is more typically associated with anthropogenic activities such as traffic emissions, industrial discharge, or domestic waste inputs. Farms from the Senegal River aligned more closely with this Cr–Pb axis, forming a compact and distinct group relative to those in Casamance. In contrast, the farms located in the Gambia River (F8–F9) clustered near the origin of the PCA space. Their proximity to the center reflects uniformly low concentrations of all analyzed PTEs, consistent with the comparatively low variability and weaker contamination gradients measured in this system. Collectively, the PCA highlighted clear and robust river-specific PTE signatures. The Casamance system is characterized by elevated concentrations of Zn, Cu, and Cd, the Senegal system by enrichment in Cr and Pb, and the Gambia system by consistently low PTE levels.

**Fig. 6:**
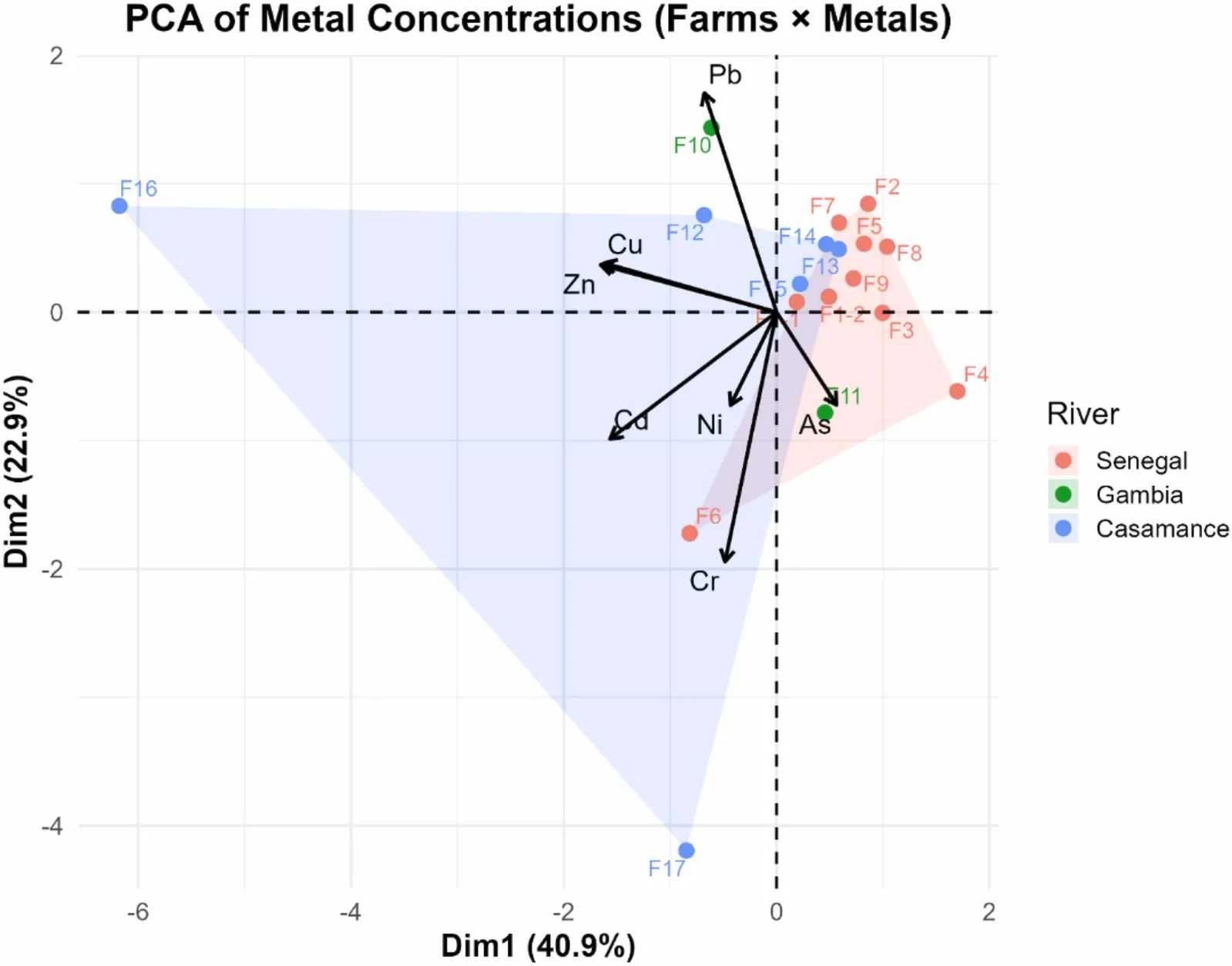
PCA biplot showing the distribution of aquaculture farms along the first two PCs based on dissolved PTE concentrations.

### 3.4 Strengths and limitations of the study

This study provides one of the first comprehensive assessments of PTEs in freshwater resources supporting aquaculture across Senegal's three major river basins. It offers a holistic evaluation of water quality, potential contamination sources, and associated public health implications. The simultaneous assessment of both SW and GW further strengthens the study by providing a more representative overview of freshwater resources used for aquaculture under contrasting hydrological conditions. In addition, the evaluation of both non-carcinogenic and carcinogenic risks for adults and children through ingestion and dermal exposure pathways provides valuable baseline information for environmental monitoring, sustainable aquaculture management, and evidence-based decision-making in Senegal.

Nevertheless, several limitations should be considered when interpreting the results. First, the study quantified total dissolved concentrations of seven PTEs but did not determine their chemical speciation. Since the toxicity, mobility, and bioavailability of elements such as Cr and As strongly depend on their oxidation state and chemical form, the actual health risks may differ from those estimated using total concentrations. Second, the investigation represents a cross-sectional assessment conducted during a single sampling campaign and therefore does not capture seasonal or interannual variations associated with river discharge, rainfall, or changing land-use practices. Long-term monitoring is required to evaluate temporal trends and the effects of climatic variability on PTE dynamics.

Additional limitations relate to the spatial and analytical scope of the study. Although GW was included because of its importance for aquaculture during the dry season, their smaller number compared with SW samples may reduce the representativeness of GW contamination patterns across Senegal. Furthermore, the investigation focused on seven priority PTEs (As, Cd, Cr, Cu, Ni, Pb, and Zn). Other inorganic contaminants (such as Hg, Mn, Fe) were not investigated and could contribute to cumulative environmental and human health risks.

The HHRA relied on deterministic USEPA exposure models and internationally accepted toxicity reference values. While these approaches are widely adopted in environmental toxicology, they do not explicitly account for mixture toxicity, cumulative low-dose effects, or inter-individual variability in exposure and susceptibility. Moreover, the assessment was limited to dissolved PTE concentrations in water and did not incorporate bioaccumulation in fish, internal biomarkers of exposure, dietary intake, or trophic transfer through aquatic food webs. Previous studies have demonstrated that these additional exposure pathways may substantially increase overall health risks in contaminated aquatic environments [[54], [55], [56], [57]].

## 4 Conclusion

This study provides the first integrated assessment of dissolved potentially toxic elements (PTEs) in freshwater resources supporting aquaculture across the Senegal, Gambia, and Casamance river basins. It provides a comprehensive evaluation of contamination patterns, potential sources, and associated public health implications. Overall, the investigated waters exhibited relatively low concentrations of the seven analyzed PTEs, with most values remaining below international drinking-water guideline values. Nevertheless, distinct spatial differences were observed among river basins. The Casamance basin exhibited comparatively higher concentrations of zinc (Zn), copper (Cu), cadmium (Cd), and nickel (Ni), whereas the Senegal River was characterized by relatively greater chromium (Cr) and lead (Pb) enrichment. In contrast, the Gambia River displayed the lowest concentrations and the least spatial variability, suggesting comparatively limited anthropogenic influence.

Multivariate analysis confirmed these basin-specific contamination patterns. The first two principal components (PCs) explained 64% of the total variance and successfully discriminated the three river systems according to their characteristic PTE signatures. The first PC was primarily associated with the combined influence of Zn, Cu, and Cd, whereas the second PC reflected contrasting contributions of Cr and Pb. In agreement with these findings, the overall concentration patterns indicated that dissolved PTEs were predominantly controlled by natural lithogenic processes, with only isolated evidence of localized anthropogenic enrichment.

The HHRA demonstrated that ingestion was the dominant exposure pathway. Children consistently experienced higher exposure than adults because of their lower body weight and higher water intake relative to body mass. Despite these differences, all hazard quotient (HQ), hazard index (HI), and carcinogenic risk (CR) values remained below the United States Environmental Protection Agency (USEPA) acceptable thresholds, indicating that the investigated waters currently pose low non-carcinogenic and carcinogenic risks to exposed populations under the assessed exposure scenarios.

Although these findings indicate that freshwater resources supporting aquaculture in Senegal remain of generally good chemical quality with respect to the investigated PTEs, several limitations should be acknowledged. Consequently, the results should be considered as an important baseline rather than a definitive assessment of long-term environmental quality.

Future research should prioritize long-term seasonal monitoring, chemical speciation, probabilistic HHRA, bioaccumulation studies in aquaculture species, and advanced source apportionment techniques, including isotopic and multivariate approaches. Integrating these complementary methodologies with ecological and epidemiological investigations will improve understanding of contaminant dynamics and provide a stronger scientific basis for sustainable aquaculture development, freshwater resource management, and environmental health protection in Senegal and throughout West Africa.

## CRediT authorship contribution statement

**Safiétou Mbaye:** Visualization, Methodology, Investigation, Formal analysis, Data curation, Conceptualization. **Dani Khoury:** Writing – original draft, Visualization, Software, Formal analysis, Data curation. **Gaspard Conseil:** Writing – review & editing, Validation, Methodology, Investigation. **Jean Fall:** Supervision, Project administration, Conceptualization. **Pamela Hartmeyer:** Investigation. **Frédéric Pierlot:** Writing – review & editing, Methodology. **Damien Banas:** Writing – review & editing, Validation, Supervision, Project administration, Conceptualization.

## Consent to publish

Not applicable

## Consent to participate

Not applicable

## Ethical approval

Not applicable

## Funding

Safiétou Mbaye received a French Cooperation Doctoral Research Mobility Fellowship (2022) to support her research stay in France. No other specific funding was received for this research.

## Declaration of Competing Interest

The authors declare no competing interests.

## Data Availability

Data will be made available on request.

## References

[1] Ali H., Khan E. (2018). Bioaccumulation of non-essential hazardous heavy metals and metalloids in freshwater fish. Risk to human health. Environ. Chem. Lett..

[2] Jaishankar M., Tseten T., Anbalagan N., Mathew B.B., Beeregowda K.N. (2014). Toxicity, mechanism and health effects of some heavy metals. Interdiscip. Toxicol..

[3] Ankapong M., Ashong G.W., Adjaottor A.A., Ababio B.A., Kwaansa-Ansah E.E., Toku M. (2025). Assessment and management of arsenic contamination in groundwater from Sanso, Ghana: Health risk implications, source identification, adsorption and immobilization for safe disposal. Toxicol. Rep..

[4] Ashong G.W., Adu Ababio B., Kwaansa-Ansah E.E., Appiah-Kubi J.A. (2025). Occurrence, metallo-toxicological interactions, and health risks of selected potentially toxic elements in the River Offin, Ghana. Sci. Rep..

[5] UNEP (2024).

[6] Ashong G.W., Kwaansa-Ansah E.E., Ababio B.A., Yagra E.A., Antwi G. (2025). Pollution profiling and quality assessment of Bonsa River, Tarkwa Nsuaem, Ghana; toxic element, ecotoxicology, health risk assessment, and multivariate analysis. Environ. Chall..

[7] Alhassan M., Ashong G.W., Ababio B.A., Kwaansa–Ansah E.E. (2025). Butachlor contamination in surface water and sediment: Assessing human health, ecological risks, and environmental implications of the Bontanga irrigation scheme in the Northern region of Ghana. Environ. Nanotechnol. Monit. Manag..

[8] Carvalho L., Figueira P., Monteiro R., Reis A.T., Almeida J., Catry T., Lourenço P.M., Catry P., Barbosa C., Catry I., Pereira E. (2018). Major, minor, trace and rare earth elements in sediments of the Bijagós archipelago, Guinea-Bissau. Mar. Pollut. Bull..

[9] Diop C. (2014). Etude de la contamination, de la spéciation et de la biodisponibilité des éléments traces métalliques dans les eaux et sédiments côtiers et estuariens au Sénégal. Eval. De. la toxicité potentielle (Dr. Diss. Lille 1).

[10] Diop C., Dewaelé D., Cazier F., Diouf A., Ouddane B. (2015). Assessment of trace metals contamination level, bioavailability and toxicity in sediments from Dakar coast and Saint Louis estuary in Senegal, West Africa. Chemosphere.

[11] Niane B., Moritz R., Guédron S., Ngom P.M., Pfeifer H.R., Mall I., Poté J. (2014). Effect of recent artisanal small-scale gold mining on the contamination of surface river sediment: Case of Gambia River, Kedougou region, southeastern Senegal. J. Geochem. Explor..

[12] Amara R., Diop M., Diop C., Ouddane B. (2019). World seas: an environmental evaluation.

[13] Ministère de la Pêche et de l’Économie Maritime (MPEM), 2023. Stratégie nationale de développement de l’aquaculture 2023–2032. Dakar, Sénégal, 71 p. Available at: https://ana.sn/wp-content/uploads/2024/05/SNDAq-2023-2032-vf2-2.pdf.

[14] Faye C. (2017). Variability and trends in observed average monthly, seasonal and annual flows in the Falémé basin, Senegal. Hydrol. Sci. J..

[15] Mohamed A., Faye C., Othman A., Abdelrady A. (2022). Hydro-geophysical evaluation of the regional variability of Senegal’s terrestrial water storage using time-variable gravity data. Remote. Sens..

[16] Gbalégba N.G.C., Silué K.D., Ba O., Ba H., Tian-Bi N.T.Y., Yapi G.Y., Kaba A., Koné B., Utzinger J., Koudou B.G. (2017). Prevalence and seasonal transmission of Schistosoma haematobium infection among school-aged children in Kaedi town, southern Mauritania. Parasit. Vectors.

[17] Pacheco L., Fraixedas S., Fernández-Llamazares Á., Estela N., Mominee R., Guallar F. (2012). Perspectives on sustainable resource conservation in community nature reserves: a case study from Senegal. Sustainability.

[18] Sow S., de Vlas S.J., Stelma F., Vereecken K., Gryseels B., Polman K. (2011). The contribution of water contact behavior to the high Schistosoma mansoni Infection rates observed in the Senegal River Basin. BMC Infect. Dis..

[19] Ahmed A.S., Hossain M.B., Babu S.O.F., Rahman M.M., Sarker M.S.I. (2021). Human health risk assessment of heavy metals in water from the subtropical river, Gomti, Bangladesh. Environ. Nanotechnol. Monit. & Manag..

[20] Shil S., Singh U.K. (2019). Health risk assessment and spatial variations of dissolved heavy metals and metalloids in a tropical river basin system. Ecol. Indic..

[21] EnHealth [Environmental Health Standing Committee] (2012). https://www.health.gov.au/sites/default/files/.

[22] USEPA (2004).

[23] USEPA, 2019. Update for Chapter 3 of the Exposure Factors Handbook. Ingestion of Water and Other Select Liquids.

[24] USEPA (1989).

[25] Li Y., Bai H., Li Y., Zhang X., Zhang L., Zhang D., Xu M., Zhang H., Lu P. (2023). An integrated approach to identify the source apportionment of potentially toxic metals in shale gas exploitation area soil, and the associated ecological and human health risks. J. Hazard. Mater..

[26] MohseniBandpi A., Eslami A., Ghaderpoori M., Shahsavani A., Jeihooni A.K., Ghaderpoury A., Alinejad A. (2018). Health risk assessment of heavy metals on PM2.5 in Tehran air, Iran. Data Brief..

[27] Jafarzadeh N., Heidari K., Meshkinian A., Kamani H., Mohammadi A.A., Conti G.O. (2022). Non-carcinogenic risk assessment of exposure to heavy metals in underground water resources in Saraven, Iran: Spatial distribution, monte-carlo simulation, sensitive analysis. Environ. Res..

[28] Adimalla N. (2020). Heavy metals contamination in urban surface soils of Medak province, India, and its risk assessment and spatial distribution. Environ. Geochem. Health.

[29] Dimowo B.O., Gbadebo A.M., Taiwo A.M., Sojinu O.S., Dimowo M.O. (2025). Carcinogenic and non-carcinogenic health risk assessment of heavy metals in water from selected oil pollution-prone communities in the Niger Delta Region. J. Trace Elem. Miner..

[30] Fahimah N., Salami I.R.S., Oginawati K., Mubiarto H. (2024). Appraisal of pollution levels and non-carcinogenic health risks associated with the emergence of heavy metals in Indonesian community water for sanitation, hygiene, and consumption. Emerg. Contam..

[31] Nazzal Y., Orm N.B., Barbulescu A., Howari F., Sharma M., Badawi A.E., A. Al-Taani A., Iqbal J., Ktaibi F.E., Xavier C.M., Dumitriu C.S. (2021). Study of atmospheric pollution and health risk assessment: A case study for the Sharjah and Ajman Emirates (UAE). Atmosphere.

[32] USEPA, 2009. Risk Assessment Guidance for Superfund (RAGS), Volume I, Human Health Evaluation Manual (Part E, Supplemental Guidance for Dermal Risk Assessment). https://www.epa.gov/risk/ risk-assessment-guidance-superfund-rags-part-f.

[33] Bruand A. (2002). Concentration and mobility of lithogenic trace metals in soils: Significance of anthropogenic lateral redistributions. Comptes Rendus Geosci..

[34] Gonzalez S., Lopez-Roldan R., Cortina J.L. (2013). Presence of metals in drinking water distribution networks due to pipe material leaching: a review. Toxicol. & Environ. Chem..

[35] Galster S., Helmreich B. (2022). Copper and zinc as roofing materials—A review on the occurrence and mitigation measures of runoff pollution. Water.

[36] Natali M., Zanella A., Rankovic A., Banas D., Cantaluppi C., Abbadie L., Lata J.C. (2016). Assessment of trace metal air pollution in Paris using slurry-TXRF analysis on cemetery mosses. Environ. Sci. Pollut. Res..

[37] Mapanda F., Mangwayana E.N., Nyamangara J., Giller K.E. (2005). The effect of long-term irrigation using wastewater on heavy metal contents of soils under vegetables in Harare, Zimbabwe. Agric. Ecosyst. & Environ..

[38] Mwegoha W.J.S., Kihampa C. (2010). Heavy metal contamination in agricultural soils and water in Dar es Salaam city, Tanzania. Afr. J. Environ. Sci. Technol..

[39] Glodji L.A., Saïzonou R., Adjo F.B., Fatiou A.K.I., Yessoufou S., Kanon B.A. (2019). Geology and Geochemistry of the Chromiferous Mineralization in the External Zone of the Pan-African Dahomeyides Belt, Northwestern Benin (Gulf of Guinea, West Africa). Int. J. Geosci..

[40] Schaefer M.V., Guo X., Gan Y., Benner S.G., Griffin A.M., Gorski C.A., Wang Y., Fendorf S. (2017). Redox controls on arsenic enrichment and release from aquifer sediments in central Yangtze River Basin. Geochim. Et. Cosmochim. Acta.

[41] WHO (2022).

[42] Gueye M., Ngom P.M., Diène M., Thiam Y., Siegesmund S., Wemmer K., Pawlig S. (2008). Intrusive rocks and tectono-metamorphic evolution of the Mako Paleoproterozoic belt (Eastern Senegal, West Africa). J. Afr. Earth Sci..

[43] Hein K.A., Morel V., Kagoné O., Kiemde F., Mayes K. (2004). Birimian lithological succession and structural evolution in the Goren segment of the Boromo–Goren Greenstone Belt, Burkina Faso. J. Afr. Earth Sci..

[44] Franken A., Eloff F.C., Du Plessis J., Du Plessis J.L. (2015). vitro permeation of metals through human skin: a review and recommendations. Chem. Res. Toxicol..

[45] Temesgen M., Alemu T., Shasho E. (2024). Heavy Metals Pollution and Potential Health Risks: The Case of the Koche River, Tatek Industrial Zone, Burayu, Ethiopia. J. Toxicol..

[46] Xue J., Zartarian V., Moya J., Freeman N., Beamer P., Black K., Tulve N., Shalat S. (2007). A meta-analysis of children’s hand-to-mouth frequency data for estimating nondietary ingestion exposure. Risk Anal..

[47] Varol M., Tokatlı C. (2022). Seasonal variations of toxic metal (loid) s in groundwater collected from an intensive agricultural area in northwestern Turkey and associated health risk assessment. Environ. Res..

[48] ATSDR [Agency for Toxic Substances and Disease Registry] (2022). https://www.atsdr.cdc.gov/spl/index.html.

[49] Eid M.H., Mikita V., Eissa M., Ramadan H.S., Mohamed E.A., Abukhadra M.R., El-Sherbeeny A.M., Bellucci S., Kovács A., Szűcs P. (2024). Monte Carlo simulation and PMF model for assessing human health risks associated with heavy metals in groundwater: a case study of the Nubian aquifer, Siwa depression, Egypt. Front. Earth Sci..

[50] Fakhri Y., Mohseni-Bandpei A., Conti G.O., Ferrante M., Cristaldi A., Jeihooni A.K., Dehkordi M.K., Alinejad A., Rasoulzadeh H., Mohseni S.M., Sarkhosh M. (2018). Systematic review and health risk assessment of arsenic and lead in the fished shrimps from the Persian gulf. Food Chem. Toxicol..

[51] Alidadi H., Tavakoly Sany S.B., Zarif Garaati Oftadeh B., Mohamad T., Shamszade H., Fakhari M. (2019). Health risk assessments of arsenic and toxic heavy metal exposure in drinking water in northeast Iran. Environ. Health Prev. Med..

[52] Moghadam S.M., Payandeh K., Koushafar A., Goosheh M., Rouzbahani M.M. (2024). Human health risk assessment and carcinogenicity due to exposure to potentially toxic elements on soil pollution in Southwest Iran. Clin. Epidemiol. Glob. Health.

[53] Ajeh E.A., Modi F.J., Omoregie I.P. (2022). Health risk estimations and geospatial mapping of trace metals in soil samples around automobile mechanic workshops in Benin city, Nigeria. Toxicol. Rep..

[54] Emurotu J.E., Olawale O., Dallatu E.M., Abubakar T.A., Umudi Q.E., Eneogwe G.O., Atumeyi A. (2024). Carcinogenic and non-carcinogenic health risk assessment of heavy metals in the offal of animals from Felele Abattoir, Lokoja, Nigeria. Toxicol. Rep..

[55] Feyisa G., Mekassa B., Merga L.B. (2025). Human health risks of heavy metals contamination of a water-soil-vegetables farmland system in Toke Kutaye of West Shewa, Ethiopia. Toxicol. Rep..

[56] Rangasamy E., Muniyandi M. (2025). Assessment of heavy metal pollution and human health risk in the soil of selected tea plantations from southern Western Ghats, Tamil Nadu, India. Toxicol. Rep..

[57] Ugwu C.E., Igbokwe A.M., Suru S.M., Dike C.C., Mbachu A.N., Maduka H.C.C. (2024). Evaluating the human health risks of heavy metal contamination in copper and steel factory effluents in Nnewi, Anambra State, Nigeria. Toxicol. Rep..

