# Supplementary material for: Occurrence, source identification, and human health risk assessment of potentially toxic elements in freshwater sources used for aquaculture in the Senegal, Gambia, and Casamance river basins
Source: Toxicol Rep. 2026 Sep 17;17:102340. doi: 10.1016/j.toxrep.2026.102340 (PMC13627819; doi:10.1016/j.toxrep.2026.102340)
Supplement: Supplementary file 1 [file mmc1.docx]

**Figure S1. Map of Senegal showing the sampling locations (Mbaye et al., 2024).**
